# Knockdown of *POLG* Mimics the Neuronal Pathology of Polymerase-γ Spectrum Disorders in Human Neurons

**DOI:** 10.3390/cells14070480

**Published:** 2025-03-22

**Authors:** Çağla Çakmak Durmaz, Felix Langerscheidt, Imra Mantey, Xinyu Xia, Hans Zempel

**Affiliations:** 1 Institute of Human Genetics, Faculty of Medicine and University Hospital Cologne, University of Cologne, 50931 Cologne, Germany; 2 Center for Molecular Medicine Cologne (CMMC), University of Cologne, 50931 Cologne, Germany; 3 Institute of Human Genetics, University Hospital Magdeburg, 39120 Magdeburg, Germany

**Keywords:** Polymerase-γ, mtDNA, SH-SY5Y, neuronal differentiation, mitochondria, neurogenetics

## Abstract

Impaired function of Polymerase-γ (Pol-γ) results in impaired replication of the mitochondrial genome (mtDNA). Pathogenic mutations in the *POLG* gene cause dysfunctional Pol-γ and dysfunctional mitochondria and are associated with a spectrum of neurogenetic disorders referred to as POLG spectrum disorders (POLG-SDs), which are characterized by neurologic dysfunction and premature death. Pathomechanistic studies and human cell models of these diseases are scarce. SH-SY5Y cells (SHC) are an easy-to-handle and low-cost human-derived neuronal cell model commonly used in neuroscientific research. Here, we aimed to study the effect of reduced Pol-γ function using stable lentivirus-based shRNA-mediated knockdown of *POLG* in SHC, in both the proliferating cells and SHC-derived neurons. *POLG* knockdown resulted in approximately 50% reductions in POLG mRNA and protein levels in naïve SHC, mimicking the residual Pol-γ activity observed in patients with common pathogenic *POLG* mutations. Knockdown cells exhibited decreased mtDNA content, reduced levels of mitochondrial-encoded proteins, and altered mitochondrial morphology and distribution. Notably, while chemical induction of mtDNA depletion via ddC could be rescued by the mitochondrial biosynthesis stimulators AICAR, cilostazol and resveratrol (but not MitoQ and formoterol) in control cells, *POLG*-knockdown cells were resistant to mitochondrial biosynthesis-mediated induction of mtDNA increase, highlighting the specificity of the model, and pathomechanistically hinting towards inefficiency of mitochondrial stimulation without sufficient Pol-γ activity. In differentiated SHC-derived human neurons, *POLG*-knockdown cells showed impaired neuronal differentiation capacity, disrupted cytoskeletal organization, and abnormal perinuclear clustering of mitochondria. In sum, our model not only recapitulates key features of POLG-SDs such as impaired mtDNA content, which cannot be rescued by mitochondrial biosynthesis stimulation, but also reduced ATP production, perinuclear clustering of mitochondria and impaired neuronal differentiation. It also offers a simple, cost-effective and human (and, as such, disease-relevant) platform for investigating disease mechanisms, one with screening potential for therapeutic approaches for POLG-related mitochondrial dysfunction in human neurons.

## 1. Introduction

The replication of the mammalian mitochondrial genome (mtDNA) depends on the heterotrimeric enzyme complex DNA polymerase γ (Pol-γ). Loss or reduced functionality of Pol-γ directly affects mtDNA synthesis and impairs mtDNA replication, which disrupts the expression of mtDNA-encoded proteins and protein subunits and leads to a constricted function of the respiratory chain and reduced provision of ATP [1,2]. The catalytic subunit of Pol-γ is encoded by the nuclear gene *POLG*. Mutations in *POLG* result in POLG-spectrum disorders, mitochondriopathies characterized by progressive mtDNA depletion, mitochondrial dysfunction and early onset neurodegeneration [3,4,5]. Clinically, the detrimental neurogenetic diseases lead to severe epilepsy, multi-organ failure and premature death (age range from 30 days to 64 years, median age 3.5 years) [5]. The two most common pathogenic POLG mutations result in the amino acid substitutions p.A467T and p.W748S. Patients with these mutations show significant residual (~5%) Pol-γ activity [5,6,7]. There is neither a cure nor an effective treatment available for POLG-SDs. Disease mechanisms are understudied, and therapeutic strategies are lacking.

The human neuroblastoma cell line SH-SY5Y is derived from the parental cell line SK-N-SH, which originates from a metastatic bone tumor biopsy [8,9]. SH-SY5Y cultures contain both epithelial-like (“S”-type) and neuroblast-like (“N”-type) cells [9,10]. “N”-type SH-SY5Y cells can be differentiated into more mature, neuron-like phenotype cells by modifying components of the cell culture medium [11]. Neuron-like SH-SY5Y cells (SHC) differentiated by treatment with retinoic acid (RA) and brain-derived neurotrophic factor (BDNF) (“SH-SY5Y-derived neurons”) constitute an established in vitro model that is commonly used to study neurodegenerative disorders such as Alzheimer’s disease [12,13] or Parkinson’s disease [14]. Besides its abilities relevant to neuronal differentiation, this cell line shows some notable advantages compared to human-induced pluripotent stem cells (hiPSCs), including ease of cultivation, rapid large-scale expansion and low costs in maintaining the culture [15,16].

The development of suitable human neuronal cell models is crucial for the in vitro mimicking of POLG-SDs in order to enable research in both the investigation of pathogenic mechanisms as well as the testing of potential treatment approaches. Still, there is a lack of cheap, easy-to-handle and established cell models for neuroscientific research of POLG-SDs, as hiPSC-based cell models are expensive, time-consuming to obtain and differentiate and often lack isogenic controls [17]. As SHC have already been used for experiments on mitochondrial biogenesis and function [18,19,20], we here test whether SHC are a suitable cell model for mitochondrial dysfunction as observed in POLG-SDs, and whether they can be a simple and easy-to-handle cell model which could be used for mimicking the phenotype in neuronal cell populations, which are the ones mostly affected. We report the generation of a stable *POLG* knockdown SH-SY5Y cell line and characterize mitochondrially based phenotypes and neuronal differentiation impairments, implying that SHC are a versatile tool for studying POLG-SD in human neurons.

## 2. Materials and Methods

### 2.1. Molecular Biology

Small hairpin RNAs (shRNAs) were cloned into pLKO.1 puro vector (Addgene #8453). For this, the following shRNA oligonucleotides were ordered and annealed: POLG sh1+ (5′-CCGGGGTGCACAGACTTTATGTACTCGAGTACATAAAGTCTGTGCACCTTTTTG-3′), POLG sh1- (5′-AATTCAAAAAGGTGCACAGACTTTATGTACTCGAGTACATAAAGTCTGTGCACC-3′), POLG sh2+ (5′-CCGGGGATGGTAATAGCTGTAATCTCGAGATTACAGCTATTACCATCCTTTTTG-3′), POLG sh2- (5′-AATTCAAAAAGGATGGTAATAGCTGTAATCTCGAGATTACAGCTATTACCATCC-3′), POLG sh3+ (5′-CCGGGCTATTACCATCCTTGTGACTCGAGTCACAAGGATGGTAATAGCTTTTTG-3′), POLG sh3- (5′-AATTCAAAAAGCTATTACCATCCTTGTGACTCGAGTCACAAGGATGGTAATAGC-3′), POLG sh4+ (5′-CCGGTTAAACTGCATTAGTAAGCCTCGAGGCTTACTAATGCAGTTTAATTTTTG-3′), POLG sh4- (5′-AATTCAAAAATTAAACTGCATTAGTAAGCCTCGAGGCTTACTAATGCAGTTTAA-3′), POLG SCR+ (5′-CCGGTTGTCTTGCATTCGACTAACTCGAGTTAGTCGAATGCAAGACAATTTTTG-3′), POLG SCR- (5′-AATTCAAAAATTGTCTTGCATTCGACTAACTCGAGTTAGTCGAATGCAAGACAA-3′).

The pLKO.1 vector was digested with abcI and defII and ligated with annealed shRNA oligonucleotides using T4 ligase (New England Biolabs GmbH, Frankfurt-Höchst, Germany). For plasmid amplification, ligation mixture was transformed into chemocompetent bacteria (*E. coli* Stbl3TM, Thermo Fisher Scientific, Langenselbold, Germany (TFS)). Bacteria were cultivated on pre-warmed agar plates with 100 μg/mL ampicillin for 16–18 h at 37 °C; single colonies were then picked and cultivated in a liquid culture (LB-Medium with 100 μg/mL ampicillin). For plasmid isolation and purification from bacterial suspensions, the PureYieldTM MidiPrep Kit (ProMega, Walldorf, Germany) was used according to the manufacturer’s protocols. Plasmid concentration and purity were checked spectrophotometrically (NanoDropTM 100, TFS). Plasmids were stored at −20 °C.

### 2.2. Production of Viral Particles

On day zero, HEK293T cells were seeded on T75 plates such that confluency would reach 70–75% on the next day. On day one, the medium on the cells was removed, the cells were washed with 1X PBS and 5 mL of DMEM was added onto cells before transfection. Transfection solution was prepared using the following vectors: 10 μg of shPOLG-plko.1 vector, 9 μg of pCMV-ΔR8.2dvpr packaging vector and 1 μg of pCMV-VSVG envelope vector. Each transfection was conducted separately for each shRNA construct. A quantity of 20 μL of PEI was added into the vector mixture and the mixture was equalized to 400 μL total volume with Opti-MEM (Gibco, TFS). The transfection mix was incubated for 20 min and added onto the cells. At 16 h post-transfection, 3 mL of DMEM was added into the plate. On day three, the medium of the cells was changed. The lentiviral particles were ready to collect after 72 h of transfection; particles in the medium were collected on days four and five. The lentiviral particles were sterilized with a 0.45 μm filter and stored at −80 °C. All the work was performed following S2-safety regulations.

### 2.3. Transfection and Selection of Knockdown Cells

SH-SY5Y neuroblastoma cells were seeded on 6-well plates, with around 30 × 10^4^ cells in each well, on day zero. The next day, the medium on the cells was removed and lentiviral particles (in DMEM) were given to the cells. On day 2, the lentiviral-particle-containing medium was removed and normal medium was given to the cells. On day 3, antibiotic selection was started and cells (including an untransfected control) were given 1 μg of puromycin in maintenance medium. Over the following days, the puromycin-containing medium was replenished every day. On day 8, when all the cells of the control group were dead, the cells successfully transduced with shRNA plasmids formed small cell colonies. Viable cells were distributed into 96-well plates in order to start a single-cell colony using the serial dilution method. These cells were observed daily, grown for about a month, and transferred into a larger dish as they grew. At the end of the single-cell colony expansion, cells were harvested and sequenced to confirm the presence of shRNA constructs.

### 2.4. SH-SY5Y Cultivation

A SH-SY5Y neuroblastoma cell line was kindly provided by Prof. Dr. Rudolf Wiesner (Institute of Veg. Physiology II, University Hospital Cologne, Cologne, Germany). SH-SY5Y cells were cultured in DMEM/F-12 GlutaMAX™ (#10565-018, TFS) supplemented with 10% fetal bovine serum (FBS, Biochrom AG, Berlin, Germany), Antibiotic–Antimycotic (1X Anti/Anti, #15240062, TFS) (referred to as ‘SHM-10’). For maintenance, SH-SY5Y cells were cultivated in uncoated T75 cell culture flasks (VWR) in a sterile incubator (Heraeus HeraCell 150, Kendro, Langenselbold, Germany) at a temperature of 37 °C, 95% air humidity and 5% CO_2_ concentration. Culture medium was changed once or twice per week, depending on cell density; the latter was monitored and images were taken with a brightfield light microscope (DM IL LED, Leica, Wetzlar, Germany), using objectives with 10× and 20× (air-based) magnification (Leica) and Las X imaging software (Leica). Cells were passaged for further cultivation or seeded for differentiation experiments at 70–80% confluency; for this, cells were washed with phosphate-buffered saline (PBS, TFS), trypsinized (0.05% Trypsin/EDTA, TFS) for 3–5 min, spun down at 1000× *g* for 3 min and resuspended in SHM-10. For passaging, the culture was diluted 1:10 and seeded again in an uncoated T75 flask. For long-term storage, trypsinized and spun-down cells were resuspended in FBS with 10% DMSO, cooled down at −1 °C/min in a cryo-container (Mr. FrostyTM, VWR, Darmstadt, Germany) and stored at −80 °C or in liquid nitrogen. For seeding, resuspended SH-SY5Y cells were counted with an automatic cell counter (TC20TM, Bio-Rad) and seeded at 3 × 10^4^ cells/cm^2^ (experiments with undifferentiated SH-SY5Y cells) or 1 × 10^4^ cells/cm^2^ (cells for differentiation with RA/BDNF) in uncoated 96-well plates, 24-well plates on glass coverslips (VWR) previously coated with 20 μg/mL Poly-D-lysine (PDL, AppliChem, Darmstadt), or uncoated 6-well plates.

### 2.5. SH-SY5Y Differentiation

Differentiation of SH-SY5Y-derived neurons was conducted using a protocol applying retinoic acid (RA) and brain-derived neurotrophic factor (BDNF), as previously reported [11]. In brief, differentiation was started 24 h after seeding at day 0 (d0). Fresh SHM-10 with 10 μM RA (Sigma-Aldrich, Taufkirchen, Germany) was added and replaced with fresh SHM-10 + 10 μM RA at d3 and d5. At d7, cells were washed once with PBS, and SHM-10 medium without FBS (referred to as ‘SHM-0’) with 10 ng/mL BDNF (Peprotech, TFS) was added and replaced with fresh SHM-0 + 10 ng/mL BDNF at d10. At d14, differentiation was completed, and cells were either fixed or harvested.

### 2.6. Measurement of Cell Viability and Drug Treatments

In order to select the correct drug concentration to administer on the SH-SY5Y cells, we conducted cell viability tests with the drugs we have tested, namely, AICAR (SelleckChem, München), cilostazol (Cayman, 1503, Hamburg, Germany), formoterol (Cayman, 15584, Hamburg, Germany), MitoQ (Cayman, 89950, Hamburg, Germany) and trans-resveratrol (Cayman, 70675, Hamburg, Germany), using a CellTiter-Glo^®^ Luminescent Cell Viability Assay (Promega, G7570, Walldorf, Deutschland). SH-SY5Y cells were seeded on 24-well plates to test for each drug and corresponding vehicle control. The following concentrations were tested on SH-SY5Y cells: AICAR in H_2_O (0.5 mM, 0.75 mM, 1 mM, 1.5 mM, 2 mM, 4 mM and 5 mM) cilostazol in DMSO (2.5 μM, 5 μM, 10 μM, 20 μM, 30 μM, 50 μM and 100 μM), formoterol in DMSO (5 nM, 10 nM, 20 nM, 30 nM, 40 nM, 50 nM and 100 nM), MitoQ in DMSO (0.25 μM, 0.5 μM, 1 μM, 2 μM, 5 μM, 10 μM and 15 μM), trans-resveratrol in DMSO (10 μM, 25 μM, 50 μM, 75 μM, 100 μM, 200 μM and 500 μM), zalcitabine (ddC) in DMSO (5 μM, 20 μM). We determined, according to the manufacturer’s instructions, the highest nontoxic concentration for each drug to be as follows: AICAR 2 mM, cilostazol 20 μM, formoterol 30 nM, MitoQ 1 μM and trans-resveratrol 75 μM. For the rescue experiment, naïve SHC were treated with 20 μM ddC for 48 h and ddC-containing medium was replenished in the middle of the treatment, at around 24 h. After ddC treatment, ddC-containing medium was removed and cells were washed 3 times with PBS. After washing, drug treatments were administered with the following concentrations: AICAR 2 mM, cilostazol 20 μM, formoterol 30 nM, MitoQ 1 μM and trans-resveratrol 75 μM.

### 2.7. Quantification of Mitochondrial DNA

In order to calculate the mtDNA amount, we used the mtDNA/nDNA ratio by using two mitochondrial DNA-encoded (MT-CTYB and MT-3319R) and two nuclear DNA-encoded (APP, 18s rRNA) genes. The total DNA was isolated from harvested cells using a QIAamp DNA kit (Qiagen, 56304, Hilden, Germany). Quantitative Real-Time PCR (RT-qPCR) was performed with SYBR green master mix. For each reaction, 6.25 μL of SYBR green mix, 4.25 μL of ddH_2_O, 1 μL and 25 ng of total DNA and 1 μL of forward and reverse primer mix (10 μM) were mixed. All reactions had 3 technique replicates. The cycling protocol for the RT-qPCR reaction was as follows: denaturation at 95 °C for 10 s, annealing at 60 °C for 20 s and elongation at 72 °C for 30 s, with 40 cycles. The relative mtDNA amount was calculated by measuring the mtDNA/nDNA ratio with the 2-ΔΔCT method.

### 2.8. Western Blot

Cells were seeded in 6-well plates, harvested with PBS and lysed with lysis buffer with 1× Phosphatase/Protease inhibitor. Protein concentrations were measured with a Bradford protein assay using Bradford Solution (AppliChem, Darmstadt, Germany) and a spectrometer (BioPhotometer RS 232 C, Eppendorf, Hamburg, Germany). For sodium dodecyl sulfate–polyacrylamide gel electrophoresis (SDS-Page), a gel casting kit (Mini-PROTEAN Tetra Cell, Bio-Rad, Dreieich, Germany) was used and 25 μg of samples (diluted in 5× Laemmli buffer) were loaded and run for 80 V for ~20 min; the current was then increased to 120 V and SDS-Page was run for approximately another 100 min until all samples had been run through the separating gel. For wet protein transfer to a polyvinylidene fluoride (PVDF) membrane (Merck, Darmstadt, Germany), the transfer was run at 30 V for ~16 h at 4 °C with constant stirring of buffer. After the transfer had finished, the transfer stack was disassembled, and the membrane was washed with Tris-buffered saline with Tween20 (TBS-T) for 1 min and then blocked for 1 h at room temperature in 5% milk in TBS-T. Membranes were incubated with the following primary antibodies for 24–48 h: anti-Pol-γ at 1:250 dilution in 5% milk in TBS-T (Santa Cruz, Heidelberg, Germany), anti-TOMM20 at 1:5000 dilution in 5% milk in TBS-T (Invitrogen, TFS), ATP5A (Mitococktail) at 1:5000 dilution in 5% milk in TBS-T (Abcam, VWR, Darmstadt, Germany) and anti-MT-COI at 1:1000 dilution in 5% milk in TBS-T (Invitrogen, TFS). After incubation with the primary antibody, the membrane was washed 3× with TBS-T (5 min each) and incubated with the corresponding secondary antibody (1:5000 dilution of anti-mouse antibody in 5% milk in TBS-T for anti-Pol-γ, ATP5A, anti-MT-COI antibodies; and 1:5000 dilution of anti-rabbit antibody in 5% milk in TBS-T for anti-TOMM20) for 1 h at room temperature. Before analysis, the membranes were washed 3× in TBS-T and incubated with ~150 μL 1:1 mixed solution of SuperSignal™ West Femto Kit (TFS). To take pictures, a Chemidoc MP Imaging System (Bio-Rad) was used, and for quantification, Image Lab 5.2.1 (Bio-Rad, Dreieich, Germany) was used.

### 2.9. ATP Measurement

ATP measurements were performed with a Perkin Elmer ATPlite Luminescence Assay System according to the manufacturer’s instructions. SH-SY5Y cells were seeded on black 96-well plates with 3 technical replicates and 40 × 10^3^ cells in each well on day 0. The next day, ATP substrate solution was added to each well at a 1:1 ratio. After 5 min of darkness adaptation, luminescence was measured with Promega Luminex Luminometer.

### 2.10. Immunofluorescence and Microscopy

Cells were cultivated as stated above in 24-well plates on glass coverslips (VWR) previously coated with 20 μg/mL Poly-D-lysine (PDL, AppliChem, Darmstadt, Germany), fixed for 30 min at room temperature in 3.7% formaldehyde (in PBS) and permeabilized + blocked with 5% bovine serum albumin (BSA, Sigma-Aldrich, Darmstadt, Germany) and 0.2% Triton X-100 (AppliChem, Darmstadt, Germany) in PBS for 10 min. Incubation with the primary antibody (the following antibodies were used: rabbit anti-Tom20 (1:500 in PBS, sc-11415, SCBT), mouse anti-ds DNA (1:1000 in PBS, ab27156, Abcam, VWR, Darmstadt, Germany) and chicken anti-MAP2 (1:2000 in PBS, ab5392, Abcam)) was conducted for 16–18 h at 4 °C. The coverslips were washed thoroughly and incubated with the corresponding secondary antibody (coupled to AlexaFluorTM fluorophores, diluted in PBS) for 1–2 h at room temperature. Nuclei were stained with 1 drop/mL NucBlueTM (Hochest 33342, TFS) for 20–30 min and samples were mounted on objective slides (#145-0011, Bio-Rad, Dreieich, Germany) with aqueous PolyMountTM (#18606, Polysciences, Hirschberg an der Bergstraße, Germany), dried for 24 at room temperature in the dark and placed in long-term storage at 4 °C in the dark.

Immunostained cells were imaged with a widefield fluorescence microscope (Axioscope 5, Zeiss, Jena, Germany), using an LED excitation lamp (Colibri 7, Zeiss, Jena, Germany), a fluorescence camera device (Axiocam 705 mono, Zeiss, Jena, Germany), a 40× (oil-based) magnification objective (Zeiss, Jena, Germany), ApoTome.2 (Zeiss, Jena, Germany) and Zen imaging software (Zen 3.6 blue edition, Zeiss, Jena, Germany). For the analysis of protein expression levels, all images were taken with identical settings (laser intensity and exposure time) to ensure statistical comparability.

## 3. Results

### 3.1. shRNA-Mediated Knockdown of POLG in Undifferentiated SH-SY5Y Cells Leads to Reduced mtDNA

**Figure 1:**
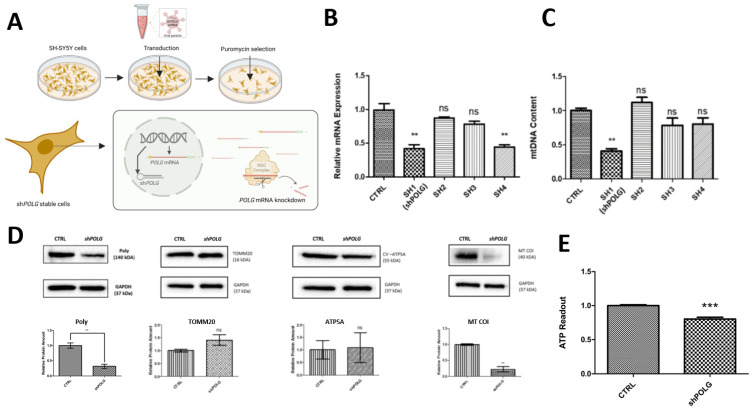
**shRNA-mediated knockdown of POLG in undifferentiated SHC leads to reduced mtDNA.** (**A**) Naïve SHC were transduced with lentiviral pLKO.1-puro vector with shRNA specifically targeting POLG. Positive colonies were selected with puromycin. (**B**) Puromycin-selected cells transduced with 4 different shRNAs show a decrease in POLG mRNA to ~50% compared to scrambled control when transduced with shRNAs 1 and 4 (SH1, SH4), but not when transduced with shRNA 2 or 3 (SH2, SH3). (**C**) Puromycin-selected SH-SY5Y cells transduced with 4 different shRNAs show a decrease of mitochondrial DNA (mtDNA) only when transduced with SH1; values decreased by ~55% percent for both mitochondrial genes MT-CYB and MT-3319R compared to the nuclear reference genes APP and 18S. (**D**) Pol-y protein expression is reduced by ~70% and the mitochondrial-encoded MT-COI protein to around 25% compared to the scrambled control, but protein levels of the nuclear-encoded outer mitochondrial membrane transporter TOMM20 and the nuclear-encoded respiratory chain component ATP5A are not significantly altered upon POLG knockdown. (**E**) ATP levels showed a 20% decrease in shPOLG cells. ** *p* < 0.001, *** *p* < 0.001, ns—not significant; experiments shown for *n* = 3–4 independent experiments.

Human neuron cell model systems are scarce in the study of rare neurogenetic disease. SH-SY5Y cells (SHC) are a commonly used in vitro model in the study of neurodegenerative disorders; they are differentiatable into neurons and potentially suitable for mimicking and studying mitochondriopathies such as POLG-spectrum disorders (POLG-SDs) [21]. To mimic dysfunction of Polymerase-γ, shRNAs were cloned into lentiviral vectors and transduced into naïve SHC to achieve RNAi-mediated knockdown of *POLG* (Figure 1A). *POLG* mRNA levels were quantified in puromycin-selected, stably transduced SHC transduced with four different shRNAs (SH1, SH2, SH3 and SH4) targeting *POLG*. The results showed that SH1 and SH4 significantly reduced POLG mRNA expression to approximately 50% compared to the scrambled control (*p* < 0.01), while SH2 and SH3 had no significant effect (Figure 1B). Additionally, mtDNA content analysis revealed that only SH1 significantly reduced mtDNA (compared to nuclear reference genes) levels, by approximately 55% compared to the scrambled control (*p* < 0.01) (Figure 1C). These findings demonstrate that SH1 effectively silences *POLG* expression at the mRNA level and efficiently reduces mtDNA content. As mtDNA encodes proteins essential for oxidative phosphorylation (OXPHOS), its proper replication is crucial for maintaining cellular energy supply. To assess the downstream effects of Pol-γ deficiency on the protein levels of mitochondrial-encoded OXPHOS components, we first confirmed that SH1 effectively silenced *POLG*, reducing its expression by 70%. Subsequently, we evaluated the protein levels of the mitochondrial-encoded core subunit of OXPHOS complex IV, Cytochrome c oxidase subunit 1 (MT-CO1). In shPOLG cells, MT-CO1 levels were significantly reduced, to approximately 25% of the control levels. In contrast, the nuclear-encoded ATP synthase subunit alpha (ATP5A) and Translocase of Outer Mitochondrial Membrane 20 (TOMM20), which facilitates the import of nuclear-encoded mitochondrial proteins, showed no significant differences in expression between control and shPOLG cells (Figure 1D). In order to test that POLG knockdown and the reduction in proteins impactenergy metabolism, we compared the ATP levels of control and shPOLG cells. ATP levels were significantly reduced, by 20% in shPOLG cells, compared to the scrambled control (Figure 1E).

**Figure 2:**
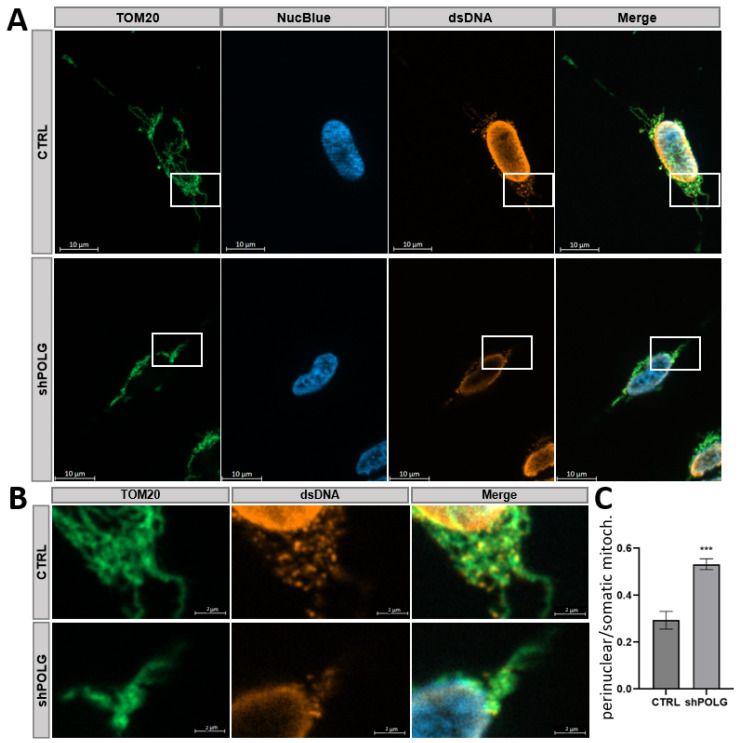
**shRNA-mediated knockdown of POLG in undifferentiated SH-SY5Y cells (SHC) leads to reduced mtDNA.** (**A**) Undifferentiated SHC were stained with TOM20 (green), NucBlue (blue) and dsDNA (orange). Control cells show the formation of a mitochondrial network with tubular, elongated mitochondria, while shPOLG cells show perinuclear localization and fragmented mitochondria. DsDNA staining intensity and colocalization with TOM20 indicates lower mtDNA content in shPOLG cells. (**B**) Magnification of boxed areas is as indicated. (**C**) Quantification of perinuclear (i.e., within 2 μM of the nuclear border) vs. whole cell mitochondria reveals a significantly increased ratio of perinuclear vs. whole cell mitochondria, indicative of perinuclear clustering of mitochondria. *** *p* < 0.001 for *n* = 3 independent experiments.

For further characterization, undifferentiated SHC were stained to detect TOM20, NucBlue and dsDNA. The control cells exhibited an interconnected network of tubular structures with elongated and filamentous mitochondria evenly distributed throughout the cell. In contrast, undifferentiated shPOLG SHC showed perinuclear clustering and appear fragmented, smaller and possibly swollen. While NucBlue stains only nuclear DNA, the anti-dsDNA antibody detects all cellular DNAs, including mtDNA. The staining intensity of dsDNA and the colocalization of non-nuclear DNA with mitochondria indicate a higher mtDNA content in control cells, compared to shPOLG knockdown cells (Figure 2A). To demonstrate this, non-nuclear DNA was visualized by subtraction of the NucBlue signal from the dsDNA and an overlay with the TOM20 signal. The resulting heatmap demonstrates a strong signal of non-nuclear DNA in proximity to mitochondria, one which also occurs in the cytoplasmic mitochondria of control cells, whereas the mtDNA signal shows spots which are fewer and more clustered in the shPOLG cells, indicating a reduction in mtDNA due to impaired mitochondrial function and dynamics resulting from the POLG knockdown (Figure 2B).

### 3.2. Stimulators of Mitochondrial Biosynthesis/Mitochondrial Protectors Reverse ddC, but Not POLG-KD-Induced mtDNA Depletion

**Figure 3:**
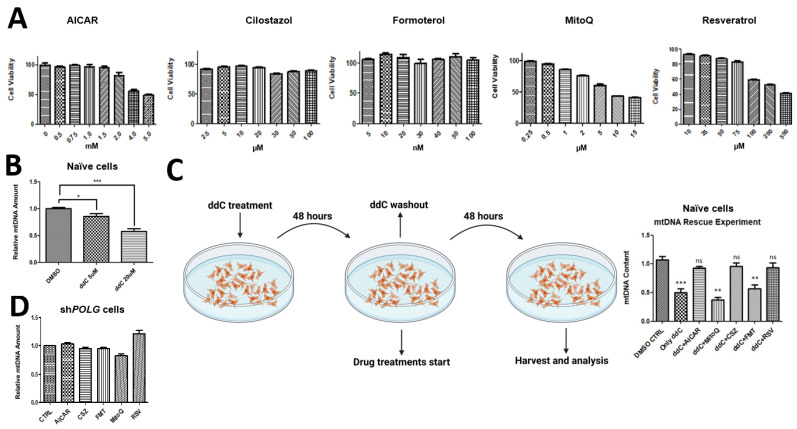
Stimulators of mitochondrial biosynthesis/mitochondrial protectors reverse ddC, but not POLG-K-induced mtDNA depletion. (**A**) Cell viability assay of mitochondrial biosynthesis inducers in SH-SY5Y cells (SHC) to identify the highest safe dose (>80% viability after 2 days of treatment, as indicated) identified 2 mM of AICAR, 20 μM of cilostazol, 30 nM, 1 μM of MitoQl and 75 μM of resveratrol as suitable doses for rescue experiments. (**B**,**C**) Chemical induction of mtDNA depletion via ddC treatment for 1 day results in a reduction in mtDNA (**B**), which can be restored by exposure for 2 days to the compounds/drugs AICAR, cilostazol(CSZ) and resveratrol(RSV), but not, or only partially by, MitoQ or formoterol (FMT), compared to control treatments. (**D**) No changes in the mtDNA in POLG-KD cells were detected when cells were exposed to mitochondrial biosynthesis stimulators/protectors, which is indicative of impaired activatability of mtDNA replication in POLG-knockdown conditions. Abbreviations: ns, not significant, * *p* < 0.05, ** *p* < 0.01, *** *p* < 0.001; for *n* = 3 independent experiments.

To investigate whether stimulators of mitochondrial biosynthesis/mitochondrial protectors can alleviate *POLG*-KD-induced mtDNA depletion, we first assessed cell viability to select the appropriate drug doses (see Methods, Section 2.6). Ultimately, we selected 2 mM AICAR, 20 μM cilostazol, 30 nM formoterol, 1 μM MitoQ and 75 μM resveratrol as the suitable doses for rescue experiments, values determined by toxicity studies (Figure 3A). Dideoxycytidine (ddC) inhibits DNA synthesis, interfering with mtDNA replication and repair. We tested whether ddC could reduce mtDNA content in SHC and found that 20 μM (but not lower doses of) ddC significantly decreased mtDNA content compared to the appropriate DMSO 0.2% control (Figure 3B). Exposure of ddC-treated cels to AICAR, cilostazol and resveratrol effectively mitigated the reduction in mtDNA content caused by ddC, whereas MitoQ and formoterol did not show appreciable effects (Figure 3B). Interestingly, the addition of mitochondrial biosynthesis stimulators/protectors to *POLG*-KD cells failed to rescue the reduction in mtDNA content (Figure 3C,D), indicating that *POLG* is essential for mtDNA.

### 3.3. shRNA-Mediated Knockdown of POLG in RA/BDNF-Differentiated SH-SY5Y Cells Results to Perinuclear Clustering of Mitochondria and Reduced Neuronal Differentiation Capacity

**Figure 4:**
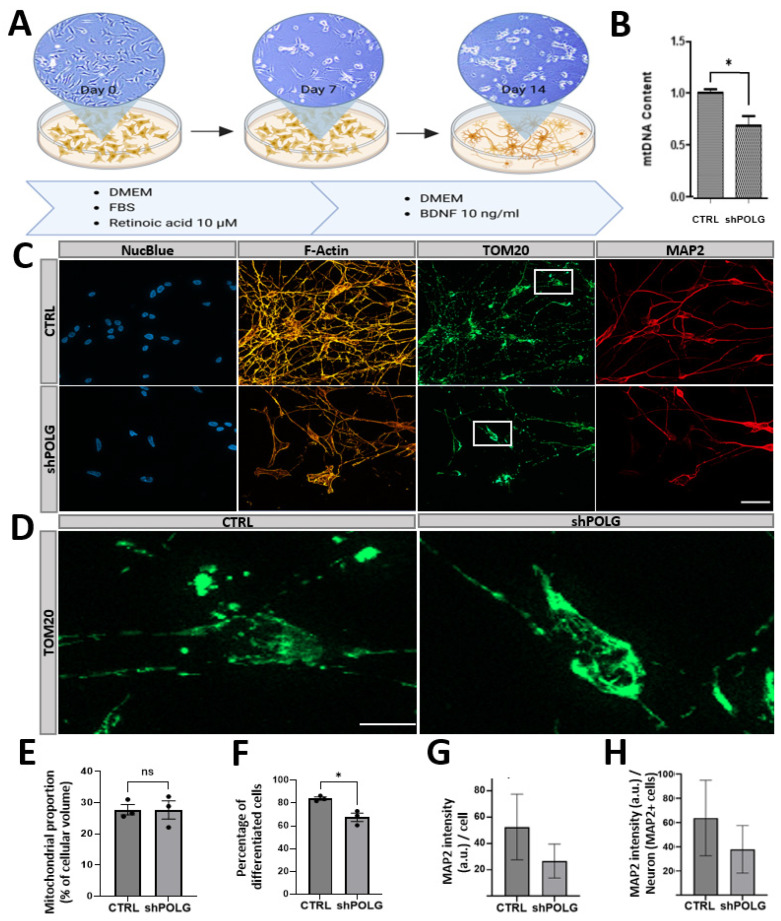
shRNA-mediated knockdown of POLG in RA/BDNF-differentiated SH-SY5Y cells results in perinuclear clustering of mitochondria and reduced neuronal differentiation capacity. (**A**) The differentiation procedure was conducted as previously reported [9,20]: naïve SH-SY5Y are treated from day 0 to day 7 with retinoic acid (RA) in DMEM with FBS. After 7 days, the medium is changed to DMEM, without FBS and with brain-derived neurotrophic factor (BDNF). After 14 days, this protocol yields > 75% neuronal cells. (**B**) The mtDNA content is lower in SH-SY5Y-derived neuronal cells with POLG knockdown than in CTRL cells. (**C**) Fluorescent staining of differentiated SH-SY5Y cells with NucBlue (blue), F-Actin (orange), TOM20 (green) and MAP2 (red). While CTRL cells are characterized by a complex F-Actin branching and cytoskeletal organization, a loss of the complex F-Actin networks can be observed in POLG-knockdown cells. TOM20 staining reveals an even distribution of mitochondria and a formation of mitochondrial networks in CTRL cells; both of which is impaired in POLG-KD cells. POLG-KD cells exhibit an accumulation of mitochondria around the nucleus (perinuclear clustering). MAP2 staining reveals that the usual neuronal morphology and prominent neurite outgrowth in RA/BDNF-differentiated SH-SY5Y cells are impaired upon POLG-KD, in line with a decreased expression of MAP2 and decreased formation of neuronal networks. Scale bar 20 μM. (**D**) Magnification of single cells stained with TOM20. Scale bar 5 μM. (**E**) Overall mitochondrial densities, calculated as the mean area of mitochondria divided by the mean area of phallodin as a total volume marker, are not significantly different in CTRL and POLG-KD cells. (**F**) Differentiation efficiency, calculated by MAP2 positive over total cells, shows impaired differentiation in POLG-KD cells. (**G**,**H**) Quantification of total MAP2 intensities normalized to the total cell count (**G**) and to the MAP2-positive cells (**H**) shows a trend towards decreased per-cell MAP2. * *p* < 0.05, 3 (**B**,**E**,**F**) or 2 (**G**,**H**) independent experiments with 5 replicates/field of views for each experiment, covering 80–150 cells.

SH-SY5Y cells (SHC) can be differentiated into more mature, neuron-like phenotype cells (SH-SY5Y-derived neurons) by modifying the components by treatment with retinoic acid (RA) and brain-derived neurotrophic factor (BDNF), providing a simple neuronal cell model for the investigation of POLG-SDs. The differentiation procedure was conducted as previously established and optimized, using a treatment with 10 μM RA for 7 days followed by 7 days with 10 ng/mL BDNF (Figure 4A). POLG-knockdown SH-SY5Y-derived neurons showed a decreased mtDNA content compared to CTRL cells, in line with our previous results from undifferentiated cells (Figure 4B). Staining of SH-SY5Y-derived neurons for NucBlue, F-actin, TOM20 and MAP2 allowed evaluation of the differentiation capacity as well as cellular morphology and mitochondrial localization. In control cells, a complex F-actin branching and cytoskeletal organization is observed, in line with a prominent neuronal morphology and neurite outgrowth indicated by the MAP2 channel. TOM20 reveals a uniform distribution of mitochondria throughout the cell. In shPOLG constructs, however, a decreased fluorescence intensity and disruption of the filament structure indicates the loss of the complex F-actin networks. The disorganized cytoskeleton correlates to a reduced neurite outgrowth and impaired development of the neuronal network, indicated by the more diffuse MAP2 expression and decreased branching (Figure 4C). Strikingly, shPOLG cells show an accumulation of mitochondria around the nucleus (white arrows, magnifications), which can be attributed to mtDNA depletion, disrupted mitochondrial dynamics, an altered microtubule network, and the induction of mitophagy (Figure 4C,D). We assessed mitochondrial density and differentiation efficiency in CTRL and POLG-KD cells. The results indicated that differentiation efficiency (MAP2-positive cells/neurons compared to total cells) was significantly reduced in POLG-KD cells, while there was only a trend toward decreased MAP2 expression per cell, whereas no significant difference was observed in overall mitochondrial density between the two groups (Figure 4E–H). In sum, POLG-KD shows less severe effects on mtDNA levels in differentiated neurons, but shows detectable impairments, both in terms of altered mitochondrial localization/somatic clustering and neuronal differentiation.

## 4. Discussion

The development of suitable neuronal cell models is crucial for investigating the pathogenic mechanisms of POLG spectrum disorders (POLG-SDs) and testing potential translatable treatments. This study presents a novel cell model for POLG-SDs using SH-SY5Y neuroblastoma cells (SHC) with stable *POLG* knockdown, which mimics key features of the disease in both naïve SHC and neuron-like states.

The shRNA-mediated knockdown of *POLG* in SHC resulted in an approximately 50% reduction in *POLG* mRNA and a 70% reduction in protein levels, which may fall short of the actual reductions in residual activity in POLG-SD when derived mutations are tested not in a cellular context, but approximates well the significant loss of enzyme activity suggested for all POLG-SDs [6]. This level of knockdown led to several phenotypes characteristic of POLG-SDs, including reduced mtDNA content, decreased levels of mitochondrial-encoded proteins, lower ATP production and altered mitochondrial morphology and distribution. Considering that *POLG*-KO cells are not viable, this knockdown approach is an excellent tool for the study of POLG-SD in human cells, without the need for cells with patient-derived mutations.

One striking finding of this study relates to the impaired neuronal differentiation capacity of *POLG*-knockdown cells upon treatment with retinoic acid (RA) and brain-derived neurotrophic factor (BDNF), which in healthy cells allows SHC to differentiate in human neurons of several lineages [21]. This observation has important implications for understanding the neurodevelopmental aspects of POLG-SDs. The reduced ability of POLG-knockdown cells to differentiate into neurons may reflect the neurodevelopmental defects observed in some POLG-SD patients with severe mutations [22], but could also suggest impaired neurogenesis once sufficient mtDNA depletion has taken place, which would be consistent with a delayed age of onset of symptoms for loss-of-functions mutations of *POLG* associated with less severe dysfunction or considerable residual activity.

Our data from differentiated *POLG*-knockdown cells suggest that mitochondrial dysfunction can significantly impact neuronal differentiation capacity, with *POLG* knockdown resulting in a significantly decreased number of neurons upon neuronal differentiation. We could not determine whether successfully differentiated cells showed significantly altered neuronal morphological features, but we did observe trends towards decreased MAP2 expression per cell and reduced complexity, which may, however, be due to reduced cell density. It is thus possible that successfully differentiated cells have found a mechanism to compensate for a reduced POLG function, which would be in line with the less severe effects on mtDNA levels observed in differentiated neurons. Noteworthy is the altered mitochondrial distribution observed in both undifferentiated and differentiated *POLG*-knockdown cells. Control cells exhibited an interconnected network of tubular mitochondria with elongated and filamentous mitochondria evenly distributed throughout the cell. In contrast, *POLG*-knockdown cells show perinuclear clustering of mitochondria which appear fragmented, smaller and possibly swollen. This perinuclear clustering, which has also been shown to play a role in other types of mitochondrial stress or dysfunction, such as oxidative stress and mitofusion impairment, may reflect disrupted mitochondrial transport along neurites—a process essential for maintaining proper neuronal function, or mitochondrial quality-control based prevention of mitochondrial trafficking into the periphery [23,24].

The study also demonstrated that *POLG*-knockdown cells are resistant to treatments that stimulate mitochondrial biosynthesis and protect against chemically induced mtDNA depletion. This resistance underscores the central role of POLG in mtDNA replication and maintenance, and suggests that therapeutic approaches for POLG-SDs may need to directly target the underlying genetic defect, rather than relying solely on general mitochondrial biogenesis stimulators. This may not hold true for POLG-SDs for which the underlying causes are mutations with considerable residual Pol-y function. There, slight stimulation may be sufficient to compensate for partial loss of enzyme function, but severe mutations will not be addressable with mitochondrial biosynthesis stimulators.

This SHC-based POLG-SD model offers several advantages for the study of these disorders. Compared to patient-derived induced pluripotent stem cells (iPSCs), SHC are easier to cultivate, expand, and maintain. The ability to differentiate SHC into neuron-like cells allows for the study of POLG-SD pathology in a more relevant cellular context, since POLG-SDs primarily affect neurons. Additionally, the use of scrambled shRNA controls provides a genetically matched comparison, reducing the variability due to genetic background differences.

This model provides valuable insights into POLG-SD pathology. As the ~50% reduction in POLG levels may not fully recapitulate the most severe cases of POLG SDs, in which there is only a little residual activity left, future studies could explore the effect of a more severe knockdown or pathogenic mutations that decrease POLG levels even more, as a complete knockout of *POLG* has been difficult to achieve, despite efforts by several laboratories, and is likely not viable. Also, we note that RA, which was used here to differentiate SHC into neurons, could enhance mitochondrial function. RA is also known to alter cellular responses to oxidative stress and mitochondrial membrane potential (MMP) in SHC, potentially attenuating the severity of the phenotype, compared to models or differentiation protocols that are not RA-based [19]. Therefore, these findings should be validated in other neuronal cell types to ensure generalizability. While the characterization we provide here is sufficient to attribute the observed effects to successful *POLG* knockdown, future studies could provide for a more in-depth characterization (oxygen consumption, extracellular acidification, mitochondrial membrane potential, electron transport chain complex activity, mitochondrial metabolite analysis, mitochondrial calcium, reactive oxygen species detection, etc.). The comprehensive data we have collected strongly support the conclusion that the observed mitochondrial impairments are due to *POLG* knockdown, and that Pol-γ dysfunction is the key factor driving the observed alterations in SH-SY5Y cells and the derived neurons. Nonetheless, future studies should also use different cell lines and investigate whether *POLG* knockdown has similar effects in other cell lines or cell types.

In conclusion, the creation of a stable *POLG* knockdown SH-SY5Y cell line represents a significant step forward in modeling POLG-SDs. By mimicking the reduced Pol-γ activity seen in patients with common *POLG* mutations, this model effectively replicates key disease features, including impaired mtDNA content, resistance to mitochondrial biosynthesis stimulation, reduced ATP production, abnormal mitochondrial distribution, and hindered neuronal differentiation. The observed resistance to rescue by mitochondrial biosynthesis stimulators in *POLG*-knockdown cells underscores the critical role of functional Pol-γ in mtDNA maintenance. Furthermore, the model’s ability to recapitulate impaired neuronal differentiation and disrupted cytoskeletal organization in differentiated neurons highlights its relevance to the neurological deficits observed in POLG-SDs. This simple, cost-effective and human-derived platform overcomes limitations found in existing models and offers a valuable tool for the dissection of disease mechanisms and the screening of potential therapeutic interventions, thereby accelerating the search for effective treatments for these devastating neurogenetic disorders. The ease of use and scalability of this model will facilitate broader research efforts aimed at understanding—and combating—POLG-related mitochondrial dysfunction in human neurons.

## Data Availability

The original contributions presented in this study are included in the article. Further inquiries can be directed to the corresponding author.

## References

[1] Cohen B.H., Chinnery P.F., Copeland W.C. (2025). POLG-Related Disorders. GeneReviews^®^ [Internet].

[2] Hance N., Ekstrand M.I., Trifunovic A. (2005). Mitochondrial DNA polymerase gamma is essential for mammalian embryogenesis. Hum. Mol. Genet..

[3] Fadic R., Johns D. (1996). Clinical Spectrum of Mitochondrial Diseases. Semin. Neurol..

[4] Finsterer J. (2004). Mitochondriopathies. Eur. J. Neurol..

[5] Zempel H., Sadzot B., Haag N. (2017). Treatment Avenues for the Juvenile and Adult Onset Mitochondriopathies Alpers Syndrome, Ataxia Neuropathy Spectrum, MEMSA and PEO Caused by Polymerase-Gamma Mutations Ala467Thr and Trp748Ser. SM J. Neurol. Neurosci..

[6] Chan S.S.L., Longley M.J., Copeland W.C. (2005). The Common A467T Mutation in the Human Mitochondrial DNA Polymerase (POLG) Compromises Catalytic Efficiency and Interaction with the Accessory Subunit. J. Biol. Chem..

[7] Luoma P.T., Luo N., Löscher W.N., Farr C.L., Horvath R., Wanschitz J., Kiechl S., Kaguni L.S., Suomalainen A. (2005). Functional defects due to spacer-region mutations of human mitochondrial DNA polymerase in a family with an ataxia-myopathy syndrome. Hum. Mol. Genet..

[8] Biedler J.L., Helson L., Spengler B.A. (1973). Morphology and growth, tumorigenicity, and cytogenetics of human neuroblastoma cells in continuous culture. Cancer Res..

[9] Kovalevich J., Langford D. (2013). Considerations for the Use of SH-SY5Y Neuroblastoma Cells in Neurobiology. Neuronal Cell Culture: Methods and Protocols.

[10] Encinas M., Iglesias M., Liu Y., Wang H., Muhaisen A., Cena V., Gallego C., Comella J.X. (2000). Sequential Treatment of SH-SY5Y Cells with Retinoic Acid and Brain-Derived Neurotrophic Factor Gives Rise to Fully Differentiated, Neurotrophic Factor-Dependent, Human Neuron-Like Cells. J. Neurochem..

[11] Langerscheidt F., Bell-Simons M., Zempel H. (2024). Differentiating SH-SY5Y Cells into Polarized Human Neurons for Studying Endogenous and Exogenous Tau Trafficking: Four Protocols to Obtain Neurons with Noradrenergic, Dopaminergic, and Cholinergic Properties. Tau Protein: Methods and Protocols.

[12] Bell M., Bachmann S., Klimek J., Langerscheidt F., Zempel H. (2021). Axonal TAU Sorting Requires the C-terminus of TAU but is Independent of ANKG and TRIM46 Enrichment at the AIS. Neuroscience.

[13] Jämsä A., Hasslund K., Cowburn R.F., Bäckström A., Vasänge M. (2004). The retinoic acid and brain-derived neurotrophic factor differentiated SH-SY5Y cell line as a model for Alzheimer’s disease-like tau phosphorylation. Biochem. Biophys. Res. Commun..

[14] Xicoy H., Wieringa B., Martens GJ M. (2017). The SH-SY5Y cell line in Parkinson’s disease research: A systematic review. Mol. Neurodegener..

[15] Yusuf M., Leung K., Morris K.J., Volpi E.V. (2013). Comprehensive cytogenomic profile of the in vitro neuronal model SH-SY5Y. Neurogenetics.

[16] Chiocchetti A.G., Haslinger D., Stein J.L., De La Torre-Ubieta L., Cocchi E., Rothämel T., Lindlar S., Waltes R., Fulda S., Geschwind D.H. (2016). Transcriptomic signatures of neuronal differentiation and their association with risk genes for autism spectrum and related neuropsychiatric disorders. Transl. Psychiatry.

[17] Cakmak C., Zempel H. (2021). A perspective on human cell models for POLG-spectrum disorders: Advantages and disadvantages of CRISPR-Cas-based vs. patient-derived iPSC models. Med. Genet..

[18] Ay M. (2022). Vanillic acid induces mitochondrial biogenesis in SH-SY5Y cells. Mol. Biol. Rep..

[19] Mu Q., Yu W., Zheng S., Shi H., Li M., Sun J., Wang D., Hou X., Liu L., Wang X. (2018). RIP140/PGC-1α axis involved in vitamin A-induced neural differentiation by increasing mitochondrial function. Artif. Cells Nanomed. Biotechnol..

[20] Hsu H.-T., Yang Y.L., Chang W.H., Fang W.Y., Huang S.H., Chou S.H., Lo Y.C. (2022). Hyperbaric Oxygen Therapy Improves Parkinson’s Disease by Promoting Mitochondrial Biogenesis via the SIRT-1/PGC-1α Pathway. Biomolecules.

[21] Bell M., Zempel H. (2022). SH-SY5Y-derived neurons: A human neuronal model system for investigating TAU sorting and neuronal subtype-specific TAU vulnerability. Rev. Neurosci..

[22] Rahman S., Copeland W.C. (2019). POLG-related disorders and their neurological manifestations. Nat. Rev. Neurol..

[23] Agarwal S., Ganesh S. (2020). Perinuclear mitochondrial clustering, increased ROS levels, and HIF1 are required for the activation of HSF1 by heat stress. J. Cell Sci..

[24] Sloat S.R., Hoppins S. (2023). A dominant negative mitofusin causes mitochondrial perinuclear clusters because of aberrant tethering. Life Sci. Alliance.

